# Influence of the Type of Macrocycle on the Stabilisation of the High Oxidation State of the Manganese Ion and Electrode Processes

**DOI:** 10.3390/molecules30081860

**Published:** 2025-04-21

**Authors:** Danuta Tomczyk, Sławomira Skrzypek, Piotr Seliger

**Affiliations:** University of Lodz, Faculty of Chemistry, Department of Inorganic and Analytical Chemistry, Tamka 12, 91-403 Lodz, Poland; piotr.seliger@chemia.uni.lodz.pl

**Keywords:** manganese dinuclear complex, cyclam, cyclen, tetraazamacrocycle, disproportionation constant

## Abstract

Dinuclear di-*µ*-oxo complexes of Mn^3+^ and Mn^4+^ ions, and mononuclear complexes of Mn^3+^ ions with tetraazamacrocycles ([12]aneN_4_, [14]aneN_4_, [15]aneN_4_) and *C*-substituted derivative (Me_6_[14]aneN_4_) as well as mononuclear complexes of Mn^2+^ ions with *N*-substituted derivatives ((*N*-Me)_2_[14]aneN_4_, (*N*-Me)_4_[14]aneN_4_, (*N*-Me)Me_2_py [14]aneN_4_) and with oxo_2_[14]aneN_4_ were studied. Based on spectroscopic (UV VIS and IR) and conductometric studies, the types of synthesised complexes (*cis* or *trans* isomers of mononuclear Mn^3+^ complexes, oxygen bridges and class II according to Robin and Day classification for dinuclear complexes) were determined. On the basis of voltammetric and spectroelectrochemical studies, *trans-cis* isomerisation at the level of Mn^2+^ ion complexes and *cis-trans* isomerisation at the level of Mn^3+^ ion complexes were demonstrated for complexes of ligands with free C positions. The *N*-substituted derivatives oxidise according to the EC mechanism, in which the follow-up reaction is a disproportionation reaction. The thermodynamic stabilisation of Mn^3+^ ions was determined by comparing the formal potentials (*E_f_*^0^), the disproportionation constants (*k*_1_) and the formation constants (*β^III^*). The study showed the possibility of oxidation of mononuclear, pseudo-octahedral Mn^3+^ ion complexes to dinuclear complexes and the greatest stabilisation of Mn^3+^ ions, both in monomers and dimers of ligands with free *N* positions.

## 1. Introduction

One of the directions for the study of manganese ion complexes results from the thermodynamic and kinetic instability of Mn^3+^ ions, which undergo disproportionation reactions to Mn^2+^ and MnO_2_ in aqueous media. Various manganese ion complexes have been studied in different aspects [1,2,3,4,5,6,7], among them azamacrocycles [8,9,10,11], which, relative to their non-cyclic counterparts, enhance the stabilisation of the thermodynamically unstable ion through a macrocyclic effect. This effect results from the superposition of entropic and enthalpic effects, associated with weaker solvation of the cyclic ligand and fewer conformational changes during complex formation.

Another direction of research of manganese ion complexes ensues from the role of this element in natural systems [12]. The structures in which it occurs in them and the way it performs certain functions are still under consideration. Therefore, complexes with geometries close to natural systems are still being studied. Their spectroscopic, structural, electrochemical and catalytic properties are being studied [13,14,15,16,17]. One of the most intensively studied natural systems is the water oxidation complex (OEC), a component of photosystem II (PS II) [18,19]. Studies of the s_0_, s_1_, s_2_ states of the OEC centre indicate a close proximity between manganese atoms with mixed valence values, 16 linear EPR signal and Robin and Day class II type compound [12,20]. Various manganese core structures are considered, most commonly dimer of dimers in which two manganese ions connected by two oxygen bridges are linked to a second dimer via an oxygen bridge and two acetate bridges [21,22]. For this reason, dinuclear complexes with oxygen or acetate bridges are being investigated as structural and functional models, in addition to the most commonly considered tetranuclear complexes [21]. Among azamacrocycles, bi-, tetra- and tri-nuclear complexes of manganese ions have been analysed, e.g., with [9]anN_3_, (*N*-Me)_3_[9]anN_3_, [12]aneN_4_ and [14]aneN_4_ [21,22].

**Scheme 1:**
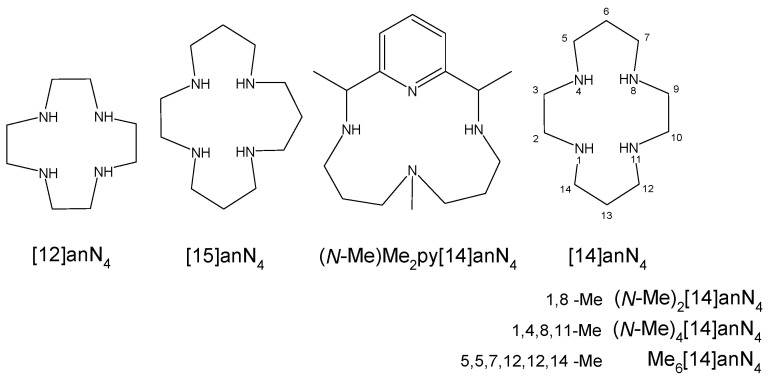
Structural formulas of the studied macrocycles.

Our choice of [14]aneN4 derivatives (Scheme 1) was due to the fact that the dinuclear complex of Mn^3+^ and Mn^4+^ ions with [14]aneN_4_, described in the literature as a structural model of the s_2_ state, was considered not only because of its typical features [23], but due to atypical characteristics such as dimer formation even from dilute solutions, *cis*-pseudo-octahedral coordination of ligands, H_2_O as a source of oxygen bridges [24]. The selection of substituents at different ligand positions allowed us to test these unusual features in the complexes they formed and to evaluate the effect of the substituents on the stabilisation of thermodynamically unstable Mn^3+^ ions in aqueous solutions. Studies conducted for complexes with [12]aneN_4_, [14]aneN_4_ and [15]aneN_4_ (Scheme 1) allowed us to analyse the effect of the size of the coordination cavity on the considered stabilisation process in water and to relate this effect to literature data for manganese complexes with [12]aneN_4_ and [14]aneN_4_ in acetonitrile [24,25]. We determined the degree of stabilisation by comparing the values of the formation constants and the formal potentials and disproportionation constants determined from electrochemical and spectroscopic analysis of the pH-dependent mechanisms of the electrode processes of the solutions.

## 2. Results and Discussion

### 2.1. Selected Aspects of the Synthesis of Complexes

The synthesis procedures used yielded different types of complexes, depending on the nature of the ligand and of the Mn^2+^ ion salt, as determined by spectroscopic studies.

**Scheme 2:**
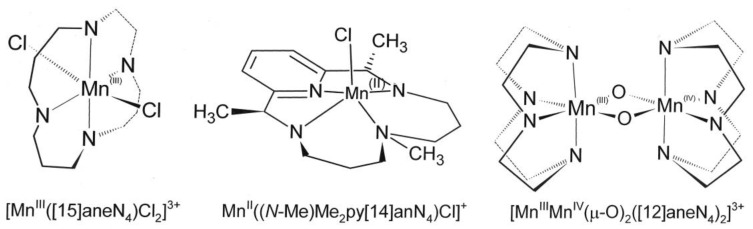
Structural formulas of the obtained complexes.

All unsubstituted and *C*-substituted ligands formed mononuclear Mn^3+^ ion complexes in acidic media (Scheme 2). The very fact that Mn^2+^ ions can be oxidised without the use of an additional oxidant and that stable mononuclear complexes of Mn^3+^ ions and their stable solutions are obtained in neutral medium testify about the significant stabilisation of the high oxidation state by these ligands.

On the other hand, in an inert medium, all these ligands show a notable tendency to form dinuclear complexes of Mn^3+^ and Mn^4+^ ions in an air atmosphere, also without the use of an additional oxidant and regardless of the Mn^2+^ ion salt used (Scheme 2). The source of the oxygen bridges is water [24]. Syntheses carried out in aqueous-alcohol solvents of various ratios have shown that for these ligands, even a minimal amount of water is sufficient to form double-core complexes. Moreover, they are formed even in very dilute solutions (10^−3^ mol·dm^−3^). Typically, higher concentrations, at least 10^−1^ mol·dm^−3^ [24], are required to obtain multicore metal ion complexes. In addition to these, mononuclear complexes of Mn^3+^ and MnO_2_ ions may be present in the reaction mixture. Therefore, to obtain only dinuclear complexes, Mn(ClO_4_)_2_, containing a non-coordinating counter ion, was used, which, with a small number of water molecules and an unusual tendency to form dimers, blocks the formation of mononuclear, hexa-coordinated complexes. Complexes of Mn^3+^ and Mn^4+^ ions with [12]anN_4_ are formed most rapidly and with the highest efficiency. Oxygen bridges hydrolyse very readily in acidic solutions, which, in such an environment, with Cl^−^ ions undergoing coordination, allowed mononuclear, hexa-coordinated Mn^3+^ complexes to be obtained. [12]anN_4_ forms a pink complex with a *cis*-octahedral structure. For the other unsubstituted and *C*-substituted ligands, the pink-red Mn^3+^ ion complexes with *cis*-octahedral structures are transitional forms that transform into light green Mn^3+^ ion complexes with *trans*-octahedral structures. Thus, *cis-trans* isomerisation is observed at the level of Mn^3+^ ion complexes. The *cis*-[Mn^III^([12]aneN_4_)Cl_2_]Cl complex does not isomerise, most likely due to the size of the coordination cavity of the macrocycle. It is the smallest among the investigated ligands and may be too small for the Mn^3+^ ion to enter the space defined by this cavity and form a *trans*-pseudo-octahedral complex in which the central atom would be in the plane of the four N atoms. Literature data show that even with a Co^3+^ ion, smaller than the Mn^3+^ ion, [12]anN_4_ forms only *cis*-pseudo-octahedral complexes [26]. This result of the synthesis of a mononuclear complex explains the reason for the remarkable ease with which [12]anN_4_ forms a dinuclear complex, in which the ligands are held by the manganese cation in *cis*-pseudo-octahedral coordination. The isomerisation process for the *cis*- [Mn^III^([15]aneN_4_)Cl_2_]Cl complex occurs after a longer time, ~1 min, which may be due to the greater flexibility of the macrocycle ring caused by the larger size of the coordination cavity and, thus, may facilitate the adoption of a conformation suitable for *cis* coordination.

A completely different synthesis result was obtained by applying this procedure to *N*-substituted and oxo_2_[14]anN_4_ derivatives. In the presence of these ligands, Mn^2+^ ions do not oxidise and, therefore, neither do mixed-valence dinuclear complexes. Mononuclear Mn^2+^ complexes are formed, which do not change their structure regardless of the pH of the solution (Scheme 2). These ligands do not form di-*μ*-oxo dinuclear complexes with Mn^2+^ ions, most likely due to the steric effect of the substituents and the limited flexibility of the macrocycle caused by the presence of a pyridine ring in the ligand structure, as well as the presence of tertiary and amide N atoms. These groups most likely hinder the macrocycle from adopting a conformation that allows the formation of the *cis*-pseudo-octahedral monomer structures necessary for the formation of a dimer of the type [Mn^III^Mn^IV^(*μ*-O)_2_L_2_]^3+^, in which the N atoms form two equatorial and two axial bonds with the manganese ions each (L stands for ligand).

Based on the syntheses carried out in the presence of the investigated ligands, it can be seen that oxidation of the Mn^2+^ ion can occur both when access to the coordination cavity of the macrocycle is unrestricted and when the substituents do not hinder the adoption of a conformation that allows the formation of the *cis*-pseudo-octahedral structure of the complex.

### 2.2. Conductometric Investigations

Molar conductivity studies were conducted in ethanol (Et), acetonitrile (AN) and water. The choice of solvents with negligible coordinating properties and lower dielectric permeability constants than water was aimed at obtaining conditions that would enable the structure of the complexes in solutions to be preserved unchanged as far as possible, relative to this structure in the solid state. The results obtained for mononuclear solutions of the complexes in Et (36.2–42.7 S·cm^2^·mol^−1^) and in AN (128.4–145.2 S·cm^2^·mol^−1^), with the exception of the complex with oxo_2_[14]aneN_4_, are within the reference ranges for 1:1 type electrolytes (35–45 in Et, 120–160 S·cm^2^·mol^−1^ in AN) [11,27]. In the case of Mn^3+^ ion complexes, these values correspond to hexa-coordinated complexes with [14]aneN_4_, Me_6_[14]aneN_4_, [12]aneN_4_ and [15]aneN_4_, which contain Cl^−^ ions at two positions. In contrast, for Mn^2+^ ion complexes, the values indicate penta-coordinated structures, [Mn^II^((*N*-Me)Me_2_py [14]aneN_4_)Cl]Cl, [Mn^II^((*N*-Me)_2_[14]aneN_4_)Cl]Cl·and [Mn^II^((*N*-Me)_4_[14]aneN_4_)Cl]Cl, in which only one position is occupied by Cl^−^ ions. For the complex of the Mn^2+^ ion with oxo_2_[14]aneN_4_, both in Et and AN, values of molar conductivities close to zero indicate a non-electrolyte and, thus, a hexa-coordinate complex, [Mn^II^(oxo_2_[14]aneN_4_)Cl_2_]. The molar conductivity values of the solutions of the dinuclear complexes (115.0–124.3 S·cm^2^·mol^−1^ in Et, 368.4–414.5 in AN) are within the reference range for electrolytes of the 3:1 type (110–130 S·cm^2^·mol^−1^ in Et, 340–420 S·cm^2^·mol^−1^ in AN) [11,27] and, thus, correspond to complexes of Mn^3+^ and Mn^4+^ ions with two oxygen bridges. In contrast, conductivity studies conducted in water for single-core Mn^3+^ ion complexes with [14]aneN_4_, Me_6_[14]aneN_4_, [12]aneN_4_ and [15]aneN_4_ showed values in the range of 418.2–431.2 S·cm^2^·mol^−1^, falling within the reference range for electrolytes of the 3:1 type (408–435 S·cm^2^·mol^−1^) [11], thus indicating an exchange of Cl^−^ ligands for water molecules, resulting from the labile nature of these complexes and, thus, complexes of the type [Mn^III^(L)(H_2_O)_2_]Cl_3_.

### 2.3. Spectroscopy

#### 2.3.1. UV/VIS/NIR Spectroscopy of Mononuclear Complexes

On the UV/VIS/NIR spectra of the aqueous solutions of the ligands (with the exception of (*N*-Me)Me_2_py [14]aneN_4_) and the complexes of Mn^2+^ ions with (*N*-Me)_2_[14]aneN_4_, (*N*-Me)_4_[14]aneN_4_ and oxo_2_[14]aneN_4_ (Figure S1, yellow and blue lines, ESI), no bands are observed but only a slightly increased absorption in the UV range (mainly for the complexes). In contrast, the spectrum of (*N*-Me)Me_2_py [14]aneN_4_ (Figure S1, red line, ESI) and its complex with Mn^2+^ (Figure S1, black line, ESI) shows absorption bands that are the result of a bathochromic shift associated with the pyridine group of the macrocycle moiety. In the case of this ligand, the bands observed after complexation are shifted to a greater extent, which may indicate greater thermodynamic stability of this complex.

**Figure 1:**
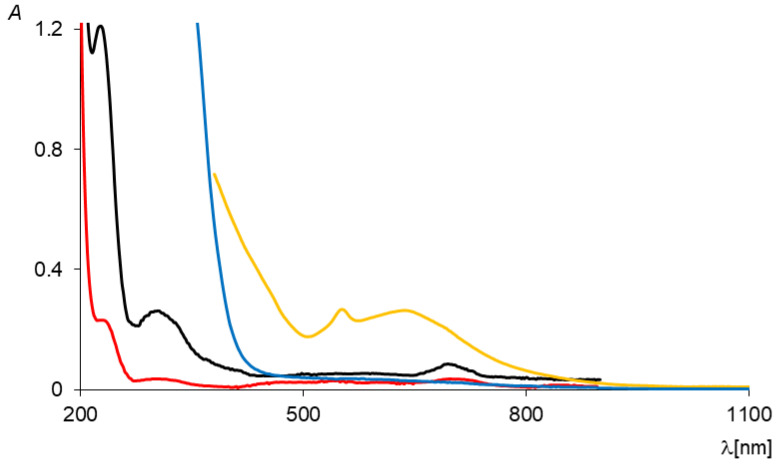
UV VIS NIR spectra of 10^−3^ mol·dm^−3^ *cis*-[Mn^III^([12]aneN_4_)Cl_2_]Cl in 0.1 mol·dm^−3^ KCl; before electrolysis, 0.093 cm cell—black line; 0.995 cm cell—blue line; during electrolysis with controlled working electrode potential at 0.4 V (80%), 0.093 cm cell—red line; 0.995 cm cell yellow line.

The spectrum of an aqueous solution of mononuclear Mn^3+^ complex with [12]aneN_4_ (Figure 1, black and blue lines) is characterised by bands at 228 (14,580), 304 (4300) and a small band at 698 nm. The absorption bands of aqueous solutions of mononuclear Mn^3+^ complexes with [14]aneN_4_, Me_6_[14]aneN_4_ and [15]aneN_4_ (Table S1, ESI) correspond to those reported in the literature, for acetonitrile solutions of these complexes [28,29,30], indicating a deformed *trans*-pseudo-octahedral geometry of the ligand field in the coordination space of the manganese ion in the third oxidation state, i.e., an elongated tetragonal bipyramid. This is evidenced by bands at the borderline of the near ultraviolet and visible ranges, at 342–398 nm, and weak bands in the 700–712 nm range, corresponding to spin-allowed *d*-*d* transitions in the Mn^3+^ ion, in a high-spin configuration. The molar absorption coefficients corresponding to the maximum absorption of the *d*-*d* transitions (*ε*_2_) are in the range ~2000–4000 dm^3^·mol^−1^·cm^−1^. Smaller than the others, the value of *ε*_2_ (760 dm^3^·mol^−1^·cm^−1^) in the *trans*-[Mn^III^Me_6_[14]aneN_4_)Cl_2_]Cl complex may be related to the steric effect of the six methyl substituents of the ligand [29]. The absorption bands occurring in the UV range, at wavelengths of 271–312 nm, with the largest *ε*_1_ values are indicative of LMCT transitions (Table S1, ESI).

In the *trans*-[Mn^III^([15]aneN_4_)Cl_2_]Cl complex (Table S1, ESI), the absorption bands originating from the *d*-*d* transitions are shifted toward longer wavelengths, most likely due to the asymmetric coordination of the Mn^3+^ ion in the xy plane, by a ligand with less symmetry than [14]aneN_4_. This translates into a higher energy d_xy_ orbital and a lower $d_{x^{2}-y^{2}}$ orbital and, therefore, a lower energy difference between the B_2g_ and B_1g_ levels, resulting in an increased wavelength *λ*_2_, responsible for the ^5^B_1g_ → ^5^B_2g_ and ^5^B_1g_ → ^5^E_g_ electron transitions in the *trans* pseudo-octahedral complex. The reduced symmetry in the Mn^3+^ coordination sphere in the *trans*-[Mn^III^([15]aneN_4_)Cl_2_]Cl complex relative to the *trans*-[Mn^III^([14]aneN_4_)Cl_2_]Cl complex is also reflected in the higher *ε*_2_ value, which is also characteristic for complexes of other transition metal ions [31].

In contrast, the shift in the absorption bands on the electron spectrum of the pink solution of the *cis*-[Mn^III^([12]aneN_4_)Cl_2_]Cl complex towards shorter wavelengths (304 nm), relative to those analysed above, suggests *cis*-pseudo-octahedral coordination of the central ion, which has been observed for other hexa-coordinated transition metal ion complexes that are *trans* and *cis* isomers [32]. Bands originating from *d*-*d* transitions in complexes with *cis*-pseudo-octahedral structures are also characterised by higher values of molar absorption coefficients [32]. The higher value of *ε_2_* of the *cis*-[Mn^III^([12]aneN_4_)Cl_2_]Cl complex (4300 dm^3^·mol^−1^·cm^−1^) compared to the *trans*-[Mn^III^([14]aneN_4_)Cl_2_]Cl complex (1980 dm^3^·mol^−1^·cm^−1^) may be related to the absence of a centre of symmetry in the *cis*-pseudo-octahedral structure.

#### 2.3.2. UV/VIS/NIR Spectroscopy of Dinuclear Complexes

**Figure 2:**
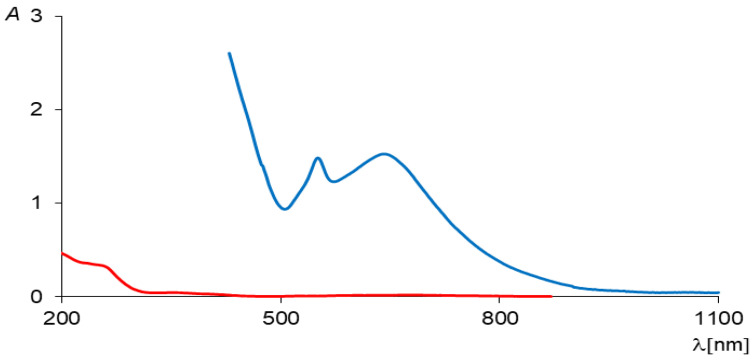
UV VIS NIR spectra of 10^−3^ mol·dm^−3^ [Mn^III^Mn^IV^(*μ*-O)_2_Me_6_[14]aneN_4_)_2_](ClO_4_)_3_ in H_2_O; blue line—0.995 cm cell, red line—0.093 cm cell.

The VIS spectra of the aqueous solutions of dinuclear Mn^3+^ and Mn^4+^ complexes with Me_6_[14]aneN_4_ and [15]aneN_4_ (Figure 2, Table S2, ESI) are very similar to acetonitrile solutions of dinuclear Mn^3+^ and Mn^4+^ complexes with [14]aneN_4_ and [12]aneN_4_ presented in the literature [24,25,33], indicating the presence of dimers in which central ions are connected by oxygen bridges. The multi-nuclear geometry of these complexes is indicated by broad bands observed in the low-energy NIR range, corresponding to transitions between metal atoms at different oxidation states. This is a characteristic feature of the Robin and Day class II compounds [12]. Bands at *λ*_3_ wavelengths (Table S2, ESI) and their intensity indicate LMCT Mn(IV) transitions. Such transitions have been observed in mononuclear complexes [34]. The bands at *λ*_2_, by analogy with the bands of complexes with Schiff bases [35], can be attributed to *d*-*d* transitions in Mn^4+^ ions. In contrast, the inflections at *λ*_1_, by analogy with the spectra of di-*μ*-oxo Mn^3+^ and Mn^4+^ complexes with bipyridine [36] indicate *d*-*d* transitions in Mn^3+^, in dinuclear complexes with mixed valence. In the 300–400 nm range, bands corresponding to transitions characteristic of *d*-*d* transitions in the Mn^3+^ ion in a mononuclear complex are absent. Only a band in UV below 300 nm is visible, indicating LMCT transitions [24].

#### 2.3.3. IR Spectroscopy of Complexes

On the IR spectra of macrocycles and their complexes, bands originating from vibrations of methyl, methylene, methanylidene groups, secondary amines, amides and pyridine groups are observed (Table S3, ESI).

Analysis of the literature shows that in the range of deformation vibrations of the amine (930–840) and methylene (830–790) groups, the complexes exhibit different numbers of bands, depending on the symmetry of the complex [37,38,39]. However, the nature of the spectrum in this range does not depend on the type of central ion or ligand. In general, complexes with *trans* symmetry are characterised by fewer bands in the range involving vibrations of both groups (930–790). These are usually one or two bands in the range of deformation vibrations of the amine groups and one corresponding to vibrations of the methylene groups. In contrast, complexes with *cis* symmetry correspond to four or five bands in the range of deformational vibrations of the amine groups and two are associated with vibrations of the methylene groups.

**Figure 3:**
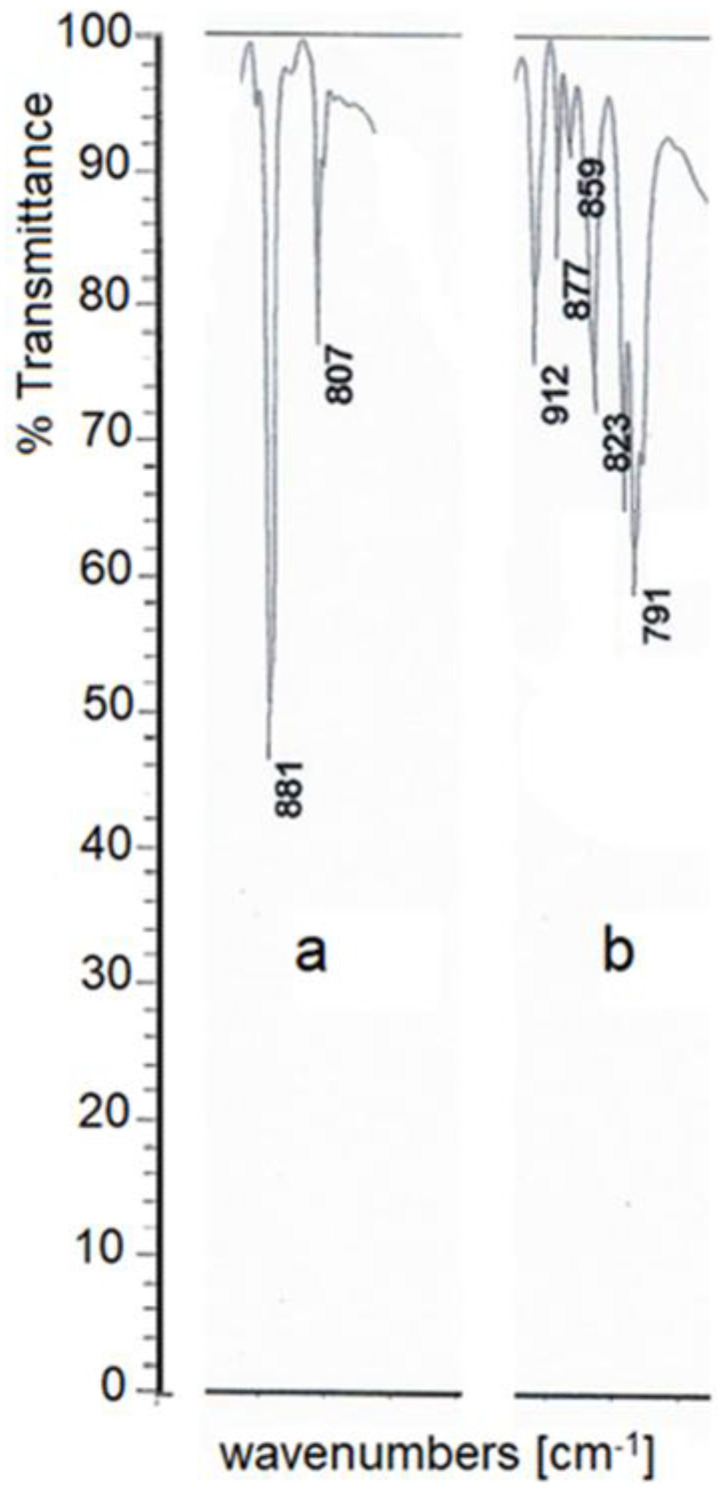
IR spectra of complexes: a—*trans*-[Mn^III^Me_6_[14]aneN_4_)Cl_2_]Cl, b—*cis*-[Mn^III^([12]aneN_4_)Cl_2_]Cl in the range of 790−930 cm^−1^, KBr pellet.

The IR spectrum of the single-core complex of the Mn^3+^ ion with Me_6_[14]aneN_4_ (Figure 3a) at 930–790 cm^−1^ is characterised by two bands, thus indicating the *trans*-pseudo-octahedral symmetry of this complex. On the IR spectrum of the complex of the Mn^3+^ ion with [15]aneN_4_ (Table S3, ESI), one band more is observed in the vibrational range of the amine groups, which may be due to the lower symmetry in the coordination sphere of this complex, resulting from the nature of the ligand.

In contrast, the IR spectrum of the Mn^3+^ ion complex with [12]aneN_4_ (Figure 3b) in the range 840–930 cm^−1^ is characterised by three bands and in the range 790–830 cm^−1^ by two bands, which, by analogy with the described classification, allows the conclusion that the structure of the analysed complex is *cis*-pseudo-octahedral.

In the spectra of the Mn^2+^ ion complexes (*N*-Me)_2_[14]aneN_4_, (*N*-Me)_4_[14]aneN_4_, oxo_2_[14]aneN_4_ and (*N*-Me)Me_2_py [14]aneN_4_ in the range of deformation vibrations of the methylene and amine groups, the number of absorption bands compared to the spectra of the corresponding ligands is the same (Table S3, ESI).

**Figure 4:**
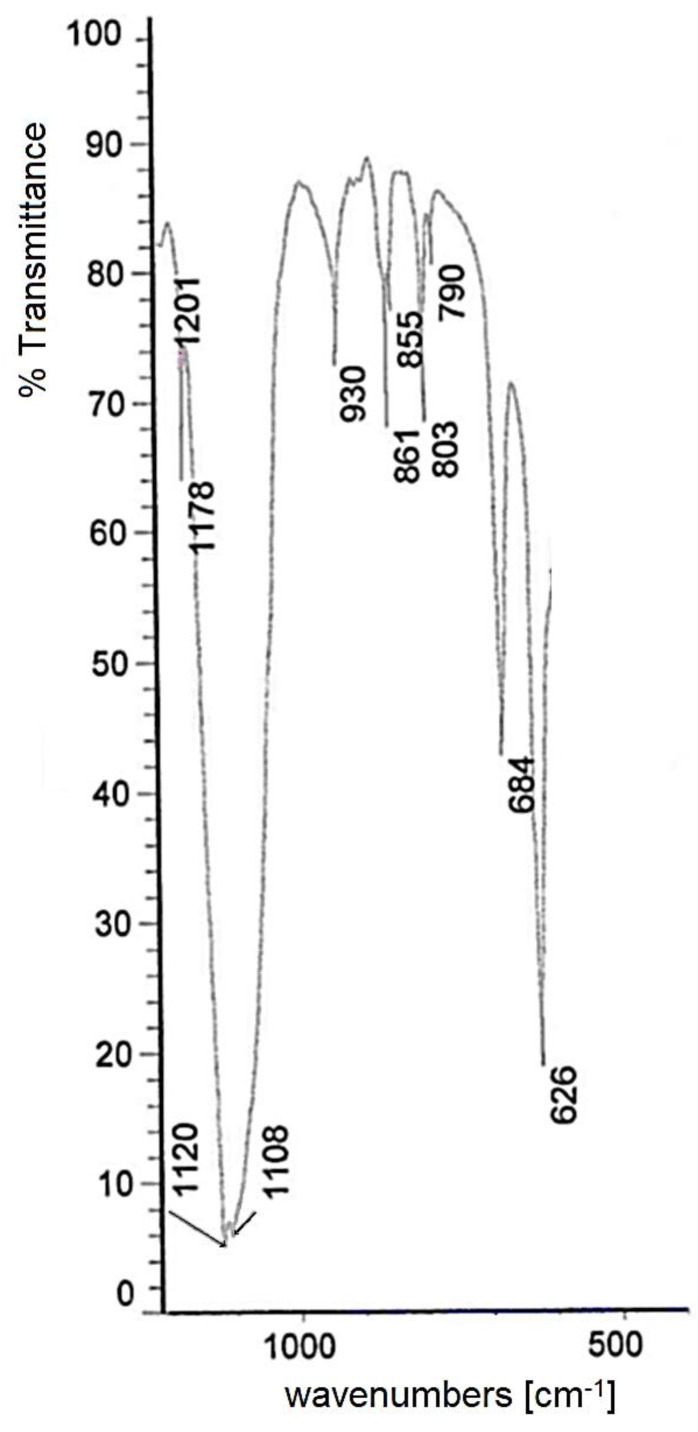
IR spectra of [Mn^III^Mn^IV^(*μ*-O)_2_Me_6_[14]aneN_4_)_2_](ClO_4_)_3_ in the range of 500−1200 cm^−1^, KBr pellet.

The IR spectra of all dinuclear Mn^3+^ and Mn^4+^ ion complexes (Figure 4, Table S4, ESI) show the same characteristics, analogous to those described in the literature for the [Mn^III^Mn^IV^(*μ*-O)_2_([14]aneN_4_)_2_]^3+^ and [Mn^III^Mn^IV^(*μ*-O)_2_([12]aneN_4_)_2_]^3+^ complexes [25,33]. The main bands occur at ~680 cm^−1^ and correspond to the vibrations of the [Mn^III^(*μ*-O)_2_Mn^IV^]^3+^ moiety [36], indicating a dinuclear di-*μ*-oxo structure of the Mn^3+^ and Mn^4+^ ion complexes with the investigated ligand. The range characteristic of the deformation vibrations of the amine and methylene groups (790–930 cm^−1^) shows an equal number of bands, two coming from the vibrations of the -CH_2_- groups and three from the vibrations of the -NH- groups. This corresponds to the structures of complexes in which the ligands adopt *cis*-pseudo-octahedral coordination with respect to the [Mn^III^(*μ*-O)_2_Mn^IV^]^3+^ moiety. The strong bands at 1120, 1108 and 626 cm^−1^ (Figure 4) originate from vibrations of ClO_4_^−^.

### 2.4. Electrochemical and Spectroelectrochemical Investigations

Spectroelectrochemical investigations were used during electrolysis runs with a controlled working electrode potential, allowing spectroscopic analysis throughout the experiments. During a given electrode process, at the potentials of the respective anodic or cathodic peaks, electron spectra were recorded at various times in the course of the process. In this way, a specific peak on the voltammogram was linked to a specific band on the electron spectrum.

Dinuclear complexes of Mn^3+^ and Mn^4+^ ions in inert medium oxidise and reduce in two steps in an irreversible and independent manner (one redox system from the other) (Figure S2, ESI), analogous to our previously described [Mn^III^Mn^IV^(*μ*-O)_2_(*iso* [14]aneN_4_)_2_](ClO_4_)_3_ complex [40], thus indicating the presence of two redox systems, [Mn^III^Mn^IV^(*μ*-O)_2_L_2_]^3+^/[Mn^III^Mn^III^(*μ*-O)_2_L_2_]^2+^ and [Mn^IV^Mn^IV^(*μ*-O)_2_L_2_]^4+^/[Mn^III^Mn^IV^(*μ*-O)_2_L_2_]^3+^. The oxidation of Mn^3+^ and Mn^4+^ ion dimers to Mn^4+^ ion dimers is confirmed by the electron spectra obtained after electrolysis with a controlled working electrode potential, carried out at the oxidation potential of the second redox system (e.g., for [Mn^III^Mn^IV^(*μ*-O)_2_([15]aneN_4_)_2_](ClO_4_)_3_ at 1 V, see Figure S2). The spectra do not show the absorbance enhancements in the VIS NIR range, characteristic of dinuclear complexes with mixed valence, corresponding to metal–metal transitions (see in Figure S3, ESI, at 800 nm, redline), nor the inflections at ~540 nm, corresponding to *d*-*d* transitions in Mn^3+^ ions. There are, however, bands associated with *d*-*d* transitions in Mn^4+^ ions (at ~550 nm) and with LMCT O-Mn(IV) transitions (at~650 nm). In contrast, the reduction of Mn^3+^ and Mn^4+^, during electrolysis at the reduction potential of the first redox system (e.g., for [Mn^III^Mn^IV^(*μ*-O)_2_([15]aneN_4_)_2_](ClO_4_)_3_ at −0.02 V, see Figure S2), to Mn^3+^ dimers is evidenced by the absence of bands in the spectra obtained after this process (Figure S3, ESI, blue line), as demonstrated in the literature for [Mn^III^Mn^III^(*μ*-O)_2_([14]aneN_4_)_2_]^2+^ in AN [24].

The mononuclear Mn^3+^ ion complexes of [12]aneN_4_, [14]aneN_4_, Me_6_[14]aneN_4_ and [15]aneN_4_ in acidic medium are reduced to Mn^2+^ ion complexes with the same symmetry, as indicated by only one signal on the voltammetric curves. For the *cis*-[Mn^III^([12]aneN_4_)Cl_2_]Cl complex, one redox system in the 0.2–0.4 V potential limit (Figure S4, ESI) corresponds to the reduction and oxidation of the *cis* structures. However, for *trans*-pseudo-octahedral Mn^3+^ ion complexes, one redox system in the −0.25 to 0 V potential limit (Figure S5, ESI) indicates redox processes occurring in *trans* structures. Considering the fact that the reduction and oxidation potentials of the *cis* and *trans* forms differ, the presence of only one redox system on the voltammetric curve confirms the presence of only one structure of a given complex. The absence of other peaks on the voltammograms during the recording of successive cycles excludes possible isomerisation during the electrode processes recorded in acidic solutions. Very weakly formed oxidation peaks in the case of complexes with *trans* structures (Figure S5, ESI) may be indicative of the instability of *trans*-pseudo-octahedral complexes of Mn^2+^ ions, what taking into account the difficulty of obtaining such complexes under current-free conditions, may be justified.

**Figure 5:**
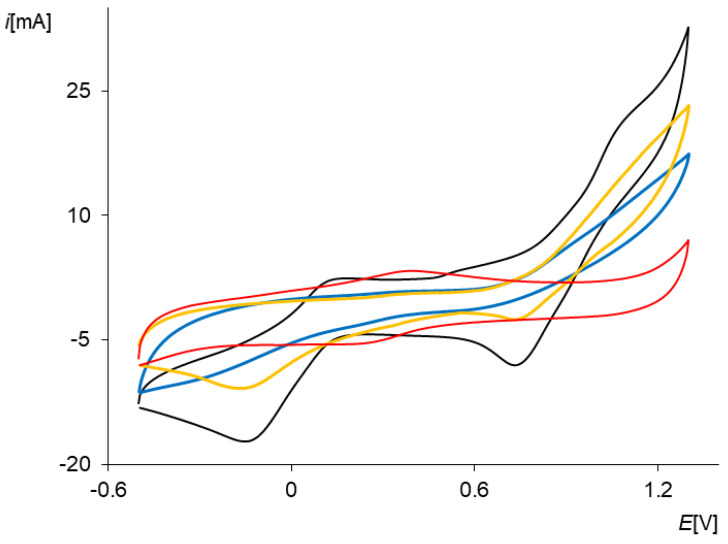
Cyclic voltammograms of 10^−3^ mol·dm^−3^ *cis*-[Mn^III^([12]aneN_4_)Cl_2_]Cl in 0.1 mol·dm^−3^ KCl; red line—1st cycle; blue line—3rd cycle; yellow line—6th cycle; black line—8th cycle; GCE vs. SCE.

In contrast, mononuclear Mn^3+^ ion complexes with [12]aneN_4_, [14]aneN_4_, Me_6_[14]aneN_4_, and [15]aneN_4_ in neutral medium, during recording of successive voltammetric curves, are oxidised to dinuclear Mn^3+^ and Mn^4+^ ion complexes, as evidenced by the obtained voltammetric curves (Figure 5, black line), analogous to those for the dimers of Mn^3+^ and Mn^4+^ ions (Figure S2, ESI) and electronic spectra. The voltammetric curves and electronic spectra were obtained based on spectroelectrochemical measurements.

In the case of *cis*-[Mn^III^([12]aneN_4_)Cl_2_]Cl, the voltammetric curves in the initial two cycles show only a single, weakly marked redox system (Figure 5, red line), analogous to the curve recorded in acidic medium (Figure S4, ESI), indicating reduction and oxidation of the complex to a complex of the same structure. In subsequent cycles, the formation of a two-step process is already observed (Figure 5, blue, yellow and black lines). The electron spectrum during electrolysis with a controlled working electrode potential, carried out at the oxidation potential of the first redox system (0.4 V) shows the presence of dimers of Mn^3+^ and Mn^4+^ ions (Figure 1, yellow line), with a simultaneous decrease in monomer concentration (Figure 1, red line).

The observed process of transition from the *cis* configuration of Mn^3+^ ion monomer to the di-*μ*-oxo dimer of Mn^3+^ and Mn^4+^ ions must, therefore, be preceded by the exchange of axial Cl^−^ ions for water molecules, deprotonation and dimerization. The former already occurs under current-free conditions, as shown by molar conductivity studies of an aqueous monomer solution, indicating a 3:1 type electrolyte. Dimerization, on the other hand, most likely occurs at the level of mononuclear Mn^3+^ ion complexes, as suggested in the literature for the *trans*-[Mn^III^([14]aneN_4_)Cl_2_]^+^ complex [24]. We excluded the possibility of dimerization at the level of a mixture of mononuclear Mn^3+^ complexes and Mn^4+^ complexes, due to the absence of a peak from the mononuclear Mn^4+^ ion complex. Only peaks corresponding to the following redox systems are observed on the voltammetric curve: *cis*-[Mn^III^([12]aneN_4_)Cl_2_]^+^/*cis*-[Mn^II^([12]aneN_4_)Cl_2_], [Mn^III^Mn^IV^(*μ*-O)_2_([12]aneN_4_)_2_]^3+^/[Mn^III^Mn^III^(*μ*-O)_2_([12]aneN_4_)_2_]^2+^ and [Mn^IV^Mn^IV^(*μ*-O)_2_([12]aneN_4_)_2_]^4+^/[Mn^III^Mn^IV^(*μ*-O)_2_([12]aneN_4_)_2_]^3+^.

The mode of oxidation of Mn^3+^ complexes with [14]aneN_4_, Me_6_[14]aneN_4_ and [15]aneN_4_,determined on the basis of spectroelectrochemical studies is analogous to the *trans*-[Mn^III^(*iso*[14]aneN_4_)Cl_2_]Cl complex we described earlier [40]. In the voltammetric curves, in the first cycle there is one redox system (Figure S6, red line), corresponding to the reduction in the complex with the *trans* structure to a complex with the same structure, in the second cycle two redox systems are already observed (Figure S6, blue line). From about the tenth cycle, a two-step electrode process can be seen, corresponding to the oxidation and reduction in dinuclear complexes of Mn^3+^ and Mn^4+^ ions (Figure S6, black line), whose presence is confirmed by the electron spectra of the solutions after the oxidation process, carried out at the potentials of the first redox system. In the case of the investigated complexes, the process of transition from monomers of Mn^3+^ ions with *trans* configurations to di-*μ*-oxo dimers of Mn^3+^ and Mn^4+^ ions, holding the ligands in *cis*-pseudo-octahedral coordination, must still be preceded by an additional isomerisation process. In the first cycle, recorded from 0 V, the presence of only one oxidation signal indicates the absence of any other structure of the mononuclear complex of the Mn^3+^ ion and, thus, *trans–cis* isomerisation at the level of the Mn^3+^ ion complexes does not occur. At the level of the complexes of these ions, an isomerisation process in the opposite direction takes place, as shown by the synthesis of the complexes. Only after the reduction process in the first cycle, another peak is observed on the oxidation curve of the second cycle at a potential of ~0.5 V (Figure S6, blue line), which may indicate, by analogy with the curve for *cis*-[Mn^III^([12]aneN_4_)Cl_2_]Cl (Figure 5, red line), the *cis*-pseudo-octahedral structure of the Mn^3+^ complex, which reduces at a potential of ~0.4 V. The isomerisation process, thus, occurs at the level of Mn^2+^ ion complexes. Literature studies have shown such a process in DMSO for *trans*-[Mn^III^([14]aneN_4_)Cl_2_]^+^ [37]. In contrast, the process of formation of dinuclear complexes of Mn^3+^ and Mn^4+^ ions under current conditions from mononuclear complexes with *trans*-pseudo-octahedral configurations took longer than the formation of [Mn^III^Mn^IV^(*μ*-O)_2_([12]aneN_4_)_2_], for which clear signals were observed already in the sixth cycle. This is reasonable given the *cis*-pseudo-octahedral symmetry of the mononuclear Mn^3+^ complex with [12]aneN_4_ and, thus, one less step in the pathway of formation of the mononuclear complex. Easy dimer formation is also observed under current-free conditions, as demonstrated by the synthesis and low stability of the solutions of *cis*-[Mn^III^([12]aneN_4_)Cl_2_]Cl complex. The [Mn^III^Mn^IV^(*μ*-O)_2_Me_6_[14]anN_4_)_2_]^3+^ complex took the longest time to form, which may be related to the steric effect of the six methyl groups in one ligand, making it difficult to adopt the *cis*-pseudo-octahedral conformation.

**Figure 6:**
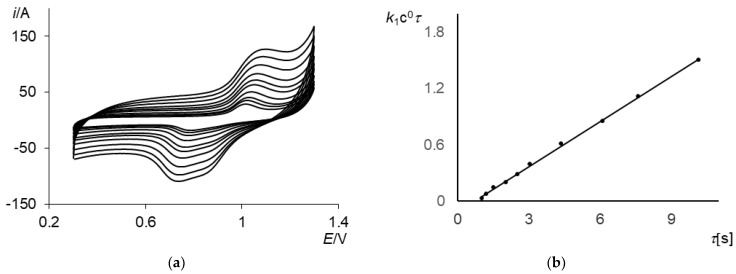
(**a**) Cyclic voltammograms of 10^−3^ mol·dm^−3^ [Mn^II^(oxo_2_[14]aneN_4_)Cl_2_] in 0.1 mol·dm^−3^ KCl; 30, 40, 50, 70, 100, 120, 150, 200, 250, 300 mV·s^−1^; GCE vs. SCE; (**b**) *k*_1_*c*^0^*t* vs. *t* plot for anodic process.

In contrast, mononuclear complexes of Mn^2+^ ions with (*N*-Me)_2_[14]aneN_4_, (*N*-Me)_4_[14]aneN_4_, (*N*-Me)Me_2_py [14]aneN_4_ and oxo_2_[14]aneN_4_ under both current and current-free conditions, do not oxidise to dinuclear complexes. In the voltammetric curves of these complexes (Figure 6a), irrespective of the pH of the solution, only one redox system is observed at potentials of 0.8–1.1 V. When recording successive cycles (at the same electrode polarisation rate v), no other anodic peaks appear, thus ruling out possible isomerisation in the −0.5–1.4 V potential range. The cathodic peaks are wider than the anodic peaks and, as the rate of electrode polarisation increases, their increasingly complex nature becomes apparent. The currents of the anodic peaks are higher than the currents of the corresponding cathodic peaks and these differences decrease as the scanning rate increases. This character of the curves indicates the EC mechanism of the electrode process, in which the subsequent reaction is a disproportionation reaction of the complexed Mn^3+^ ions [41] and, thus, a weaker stabilisation of these ions by *N*-substituted macrocycles and oxo_2_[14]aneN_4_.

The values of the disproportionation reaction rate constants (*k*_1_) were determined from voltammetric curves recorded at different electrode polarisation rates, from the slope of the *k*_1_*C*^0^*τ* = f(*τ*) relation (Figure 6b), based on the values of the kinetic parameters 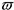 and *τ*, defined as log 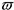 = log(*k*_1_*c*^0^*τ*) + 0.047(*ατ*-4) and *τ* = (*E_λ_* − *E*^0^*_f_*)/*v*, where *E_λ_* denotes the potential at which the electrode polarisation changes, according to the procedure presented in [42]. The obtained results (Table 1) indicate that, in this group of complexes, the Mn^3+^ ion is stabilised to the greatest extent by (*N*-Me)Me_2_py [14]aneN_4_. On the other hand, to the smallest extent by oxo_2_[14]aneN_4_, most likely due to the reduced electron density of the two amide N atoms, affecting a worse complexation effect and thus an easier disproportionation reaction. All these ligands, however, stabilise Mn^3+^ ions better than the phenanthroline derivatives we studied earlier [42]—lower *k*_1_ values for the macrocyclic complexes.

The formal potentials (*E*^0^*_f_*) of the analysed redox systems were determined at low electrode polarisation rates, using the polarisation curve method, due to the non-reversibility of the electrode processes [43]. All the values of the formal potentials of the redox systems [Mn^III^LCl_2_]^+^/[Mn^II^LCl_2_] and [Mn^III^Mn^IV^(*μ*-O)_2_L_2_]^3+^/[Mn^III^Mn^III^(*μ*-O)_2_L_2_]^2+^ are lower than the formal potential of the Mn^3+^/Mn^2+^ system and indicate stabilisation of the central ion according to macrocyclic effect, to a degree dependent on the type of the ligand (Table S5, ESI). The Mn^3+^ ion is best stabilised by mononuclear Mn^3+^ complexes with a *trans*-pseudo-octahedral structure. Formal potential values of the redox *trans*-[Mn^III^LCl_2_]^+^/*trans*-[Mn^II^LCl_2_] systems are the lowest, ranging from −0.04 to −0.16 V. Among the dinuclear complexes, the formal potentials of the first steps of the electrode processes are higher, varying between 0.01 and 0.07 V. The lowest value of the formal potential is exhibited by the [Mn^III^Mn^IV^(*μ*-O)_2_([12]aneN_4_)_2_](ClO_4_)_3_ complex, which is justified, taking into account the fact that it is this complex that is also most easily obtained under current-free conditions. For the complexes [14]aneN_4_ and its derivative, the direction of changes in the formal potential values of the Mn^III^Mn^IV^(*μ*-O)_2_L_2_]^3+^/[Mn^III^Mn^III^(*μ*-O)_2_L_2_]^2+^ system is opposite to those for the *trans*-[Mn^III^LCl_2_]^+^/*trans*-[Mn^II^LCl_2_] one. In the *trans*-[Mn^III^LCl_2_]^+^ complex, electron-donor substituents reduce the stabilisation of the high oxidation state (*E*^0^*_f_* of the *trans*-[Mn^III^Me_6_[14]aneN_4_Cl_2_]^+^/*trans*-[Mn^II^Me_6_[14]aneN_4_Cl_2_] system is higher than *E*^0^*_f_* of the *trans*-[Mn^III^[14]aneN_4_Cl_2_]^+^/*trans*-[Mn^II^[14]aneN_4_Cl_2_] system, Table S5, ESI), whereas in the [Mn^III^Mn^IV^(*μ*-O)_2_L_2_]^3+^ complex they increase the stabilisation (the *E*^0^*_f_* values for the dimers of these ligands are inversely related). The reason for the higher stabilisation of the dimers of smaller ions, Mn^4+^ and Mn^3+^, by two ligands containing electrodonor substituents linked by a manganese core into *cis*-pseudo-octahedral structures may be the lower flexibility of such a structure. The steric effects of the substituents in the case of the manganese core stiffening the structure of the complex may hinder the change to one that would allow the coordination of the larger central ions (two Mn^3+^ ions). In contrast, for the *trans*-[Mn^III^([15]anN_4_)Cl_2_]^+^/*trans*-[Mn^II^([15]anN_4_)Cl_2_] and [Mn^III^Mn^IV^(*μ*-O)_2_([15]anN_4_)_2_]^3+^/[Mn^III^Mn^III^(*μ*-O)_2_([15]anN_4_)_2_]^2+^ systems, the highest *E*^0^*_f_* values (−0.04 V and 0.07 V, respectively) may be related to the larger size of the coordination cavity of this ligand, facilitating the coordination of the larger ion. The even higher value of the formal potential of the *cis*-[Mn^III^([12]anN_4_)Cl_2_]^+^/*cis*-[Mn^II^([12]anN_4_)Cl_2_] system (*E*^0^*_f_* = 0.34 V), indicating weaker stabilisation of the Mn^3+^ ion by this ligand, is due to the different symmetry of this complex.

Significantly higher formal potential values are observed for [Mn^III^LCl]^2+^/[Mn^II^LCl]^+^, where L = (*N*-Me)_2_[14]aneN_4_, (*N*-Me)_4_[14]aneN_4,_ (*N*-Me)Me_2_py [14]aneN_4_ and [Mn^III^(oxo_2_[14]aneN_4_)Cl_2_]^+^/[Mn^II^(oxo_2_[14]aneN_4_)Cl_2_] (0.8–0.89 V) redox systems, thus indicating a worse stabilisation of the Mn^3+^ ions. The [Mn^II^((*N*-Me)Me_2_py [14]anN_4_)Cl]Cl complex is the easiest to oxidise (*E*^0^*_f_* = 0.8 V), while [Mn^II^(oxo_2_[14]anN_4_)Cl_2_] is the most difficult (*E*^0^*_f_* = 0.89 V).

### 2.5. Potentiometric Investigations

**Figure 7:**
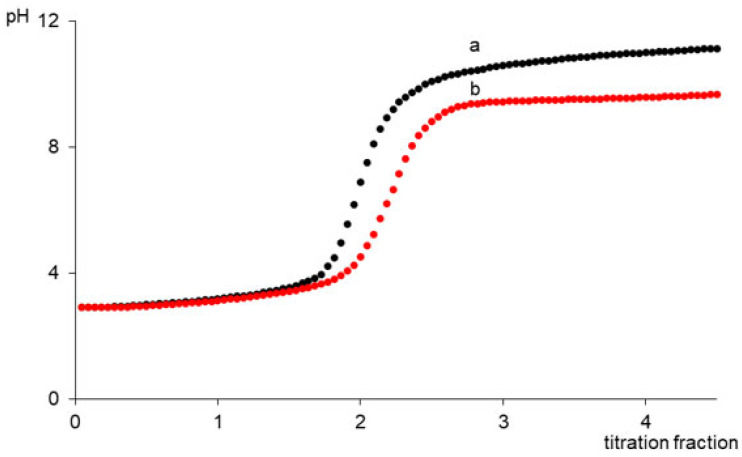
pH titration curves of the protonated (*N*-Me)_2_[14]aneN_4_ solution in the absence and presence of Mn^2+^ ions, T = 293 K, *μ* = 0.1(10^−1^ mol·dm^−3^KCl); a—(*N*-Me)_2_[14]anN_4_ (*c* = 10^−3^ mol·dm^−3^), HCl (*c* = 4·10^−3^ mol·dm^−3^); b—(*N*-Me)_2_[14]anN_4_ (*c* = 10^−3^ mol·dm^−3^), HCl (*c* = 4·10^−3^ mol·dm^−3^), MnCl_2_ (*c* = 10^−3^ mol·dm^−3^).

Equilibrium studies of the complexation of Mn^2+^ and Mn^3+^ ions were conducted to determine the thermodynamic stability of the complexes. The formation constants of manganese complexes were determined from potentiometric studies. The formation constants of Mn^2+^ complexes (*β*^II^) were determined based on pH measurements (Figure 7). Mn^3+^ complex formation constants (*β*^III^) were determined from EMF measurements of cells containing the Mn^3+^/Mn^2+^ redox system in the presence of a protonated ligand, at pH = 2 and ionic strength constant *μ* = 0.1(KCl). Mn^3+^ ions in the cell under study were produced following the addition of the MnO_4_^−^ oxidant, to a solution containing, among other species, Mn^2+^ ions. In the medium of pH = 2, in the presence of a complexing agent and a tenfold excess of Mn^2+^ ions, the reaction occurred rapidly and quantitatively and no disproportionation reaction of Mn^3+^ ions was observed. Despite the low pH, complexation of Mn^3+^ ions occurred, as evidenced by the pink solutions obtained during this procedure and the *β*^II^ values obtained (Table 1) higher than the protonation constants. The values of the protonation constants (K) of the analysed macrocycles, necessary for the determination of *β* are available in the literature, but they were also determined assuming that the same conditions of the experiment would reduce the error of the calculated *β* values.

Values of the protonation and stability constants of Mn^2+^ complexes were calculated using Hyperquad 2003 software [44]. Automating weighting scheme were used for automatic refining of the constants.

The logK values obtained (Table S6) indicate a reduction in the basicity of the macrocycles as a result of the introduction of electrodonor and electroacceptor substituents into the [14]aneN_4_ structure, which may be related to the steric effect of the substituents and the mesomeric effect of the pyridine and amide groups of the ligands.

The formation constants of the Mn^2+^ ion complexes (*β*^II^) with the studied macrocycles (Table 1) are relatively small and not significantly different, characteristic of an ion not stabilised by the ligand field. The presence of complexed Mn^2+^ ions in solutions, in the pH range studied, is indicated by the titration curve (Figure 7), the presence of such forms in the Mn^2+^/Mn^2+^L partition curves (Figure S7) and the obtained *β*^II^ values (Table 1).

Among the unsubstituted ligands, the complex with [12]aneN_4_ shows the highest value, which is most likely due to the *cis*-pseudo-octahedral structure, for which the size of the central ion is less important than in the case of *trans*-pseudo-octahedral structures. The values of the formation constants of the complexes with [14]aneN_4_ and Me_6_[14]anN_4_ are similar. This may indicate a balanced result of generally oppositely acting inductive and steric effects related to the presence of methyl substituents in *C*-substituted [14]aneN_4_.

Among the *N*-substituted and oxo_2_[14]aneN_4_ ligands, it is the latter that forms the least stable complexes. The reason for this is that the electron density of the two amide N atoms is lowered so much that they do not protonate at all in acidic solution, as shown by protonation equilibrium studies. Also, a relatively low log*β*^(II)^ value is exhibited by the complex of the Mn^2+^ ion with (*N*-Me)_4_[14]anN_4_, which may be related to its spatial structural. Literature studies show that the (*N*-Me)_4_[14]anN_4_ ligand has an undulating structure due to the *sp*^3^ hybridisation of the C and N atoms. The Mn^2+^ ion in complex with this ligand is located above the macrocycle plane, on the same side as the methyl substituents [45]. This spatial orientation may hinder the access of the central ion to the N atoms, which in effect results in a lower stability of the complex of the Mn^2+^ ion with (*N*-Me)_4_[14]anN_4_. In contrast, the lower steric effect of the substituents in the *N*-substituted, dimethyl derivative of [14]anN_4_ influences the increase in log*β*^(II)^ value. In this group of ligands, the complex of the Mn^2+^ ion with (*N*-Me)Me_2_py [14]aneN_4_ shows the highest log*β*^(II)^ value. Such a result may indicate that the mesomeric effect of the pyridine group, reducing the electron density of the ligand atom, is outweighed by the inductive effect of the methyl substituents, increasing the electron density. The two of methyl substituents in the C positions are unlikely to hinder coordination. An additional factor increasing the stability of this complex may be the weaker solvation of the ligand with less hydrophilicity, which facilitates complexation.

The determination of the values of the formation constants (*β*^III^) of Mn^3+^ ions complexes, which are always accompanied by Mn^2+^ ions in excess in solutions, based on EMF measurements, required the development of a suitable computational programme [46]. The programme was written in Delphi programming environment and is based on the PKAS [47], MINIGLASS and BEST [48] algorithms published in the literature. The input data for the programme, in addition to the concentrations and volumes of solutions, were a curve describing the dependence of the formal potential on the concentration of the oxidised form of the system, with a constant concentration of the reduced form of the redox system and constant concentrations of the ligand and hydrogen ion, as well as the ligand protonation constants and constants for the formation of its complexes with the reduced form of the system, determined in independent experiments. The next step is to select a model of the complexation process that the oxidised form of the metal ion undergoes. The model includes the maximum number of ligands that can bind to the central ion (up to 6), the maximum number of hydrogen ions that can be bound in the protonated complexes (0–3) and the maximum number of hydroxyl ions (0–3) included in the complexes. For each measurement point, a system of mass balance equations [46]. is solved on the basis of the proposed model. The equilibrium concentrations [Mn^3+^], [Mn^2+^] and [L] are solved, which allows to calculate the potential that the studied redox system should have for an assumed set of formation constants. The curve thus obtained is compared with the experimental curve (calculates the sum of squares of deviations) and corrections are made to the initial set of constants. These operations are repeated until convergence is achieved (the sum of squares of deviations of the calculated curve from the experimental curve becomes smaller than the assumed accuracy). The performance of the programme was tested on the Fe(III)/Fe(II) system, for which many values of complex formation constants are available in the literature. This system was tested in the presence of picolinic acid, oxalic acid, citric acid and nitrilotriacetic acid. The results obtained [46], coinciding with those in the literature, indicate that the measurement procedure and the calculation programme work correctly.

The values of the complex formation constants of Mn^3+^ (*β*^III^) ions with (*N*-Me)_2_[14]aneN_4_, (*N*-Me)_4_[14]aneN_4,_ oxo_2_[14]aneN_4_ and (*N*-Me)Me_2_py [14]aneN_4_ are higher than the corresponding values of log*β*^(II)^, which, in addition to the smaller radius of the central ion, is influenced by the crystal field stabilisation energy for the *d^4^* ion in the weak field of the ligands. The direction of change in the stability of Mn^3+^ ion complexes for this group of ligands is the same as the direction of change in the stability of Mn^2+^ ion complexes.

## 3. Materials and Methods

### 3.1. Ligands

1,4,8,11-tetrazacyclotetradecane ([14]aneN_4_) and its pyridine, methyl and oxo derivatives ((*N*-Me)Me_2_py [14]aneN_4_, Me_6_[14]aneN_4_, (*N*-Me)_2_[14]aneN_4_, (*N*-Me)_4_[14]aneN_4_, oxo_2_[14]aneN_4_), 1,4,7,10-tetraazacyclododecane ([12]aneN_4_) and 1,4,8,12-tetraazacyclopentadecane ([15]aneN_4_)) were from Sigma Aldrich and were used as received.

### 3.2. Complexes

#### 3.2.1. Mononuclear Complexes

Mononuclear manganese ion complexes were synthesised based on the previously described procedures [30,40,45].

The solution of MnCl_2_·4H_2_O (10^−3^ mol) in ethanol (10 cm^3^) was added dropwise to the solution of ligands (10^−3^ mol) in ethanol (10 cm^3^)—green-brown solids are formed. (In the case of (*N*-Me)_2_[14]aneN_4_, (*N*-Me)_4_[14]aneN_4,_ (*N*-Me)Me_2_py [14]aneN_4_ and oxo_2_[14]aneN_4_−white solids are formed) The reaction mixtures were left with stirring for 1.5 h at room temperature. Addition of concentrated hydrochloric acid (0.1 cm^3^) gave a red-pink solids (In the case of (*N*-Me)_2_[14]aneN_4_, (*N*-Me)_4_[14]aneN_4_, (*N*-Me)Me_2_py [14]aneN_4_ and (oxo)_2_[14]aneN_4_ white solids remained unchanged). After ~30 s red-pink solids turn into a clear green precipitate (In the case of [12]anN_4_ the precipitate remained pink). The white solids of (*N*-Me)_2_[14]aneN_4_, (*N*-Me)_4_[14]aneN_4_, (*N*-Me)Me_2_py [14]aneN_4_ and oxo_2_[14]aneN_4_ remained unchanged regardless of time. All precipitates were filtered off, washed with ethanol, recrystallized from water and dried under vacuum at room temperature. As a result of this procedure, the following complexes were obtained:

- *trans*-[Mn^III^([14]aneN_4_)Cl_2_]Cl·2H_2_O; Yield: 54%. Anal. Calcd. for C_10_H_24_N_4_Cl_3_Mn·2H_2_O: C, 30.20; H, 7.04; N, 14.09. Found C, 30.39; H, 7.00; N, 13.92;
- *trans*-[Mn^III^Me_6_[14]aneN_4_)Cl_2_]Cl·2H_2_O; Yield: 44%. Anal. Calcd. for C_16_H_36_N_4_Cl_3_Mn·2H_2_O: C, 39.89; H, 8.30; N, 11.63. Found C, 39.97; H, 8.51; N, 11.68;
- *cis*-[Mn^III^([12]aneN_4_)Cl_2_]Cl·3H_2_O; Yield: 53%. Anal. Calcd. for C_8_H_20_N_4_Cl_3_Mn·3H_2_O: C, 24.79; H, 6.71; N, 14.46. Found C, 23.65; H, 6.75; N, 14.51;
- *trans*-[Mn^III^([15]aneN_4_)Cl_2_]Cl·3H_2_O; Yield: 51%. Anal. Calcd. for C_11_H_26_N_4_Cl_3_Mn·3H_2_O: C, 30.75; H, 7.45; N, 13.04. Found C, 31.18; H, 7.57; N, 13.08;
- [Mn^II^((*N*-Me)_2_[14]aneN_4_)Cl]Cl·3H_2_O; Yield: 52%. Anal. Calcd. for C_12_H_28_N_4_Cl_2_Mn·3H_2_O: C, 35.30; H, 8.33; N, 13.73. Found C, 35.15; H, 7.99; N, 13.65;
- [Mn^II^((*N*-Me)_4_[14]aneN_4_)Cl]Cl·5H_2_O; Yield: 50%. Anal. Calcd. for C_14_H_32_N_4_Cl_2_Mn·5H_2_O: C, 35.60; H, 8.89; N, 11.87. Found C, 35.98; H, 8.63; N, 11.91;
- [Mn^II^((*N*-Me)Me_2_py[14]aneN_4_)Cl]Cl·H_2_O; Yield: 52%. Anal. Calcd. for C_16_H_29_N_4_Cl_2_Mn·H_2_O: C, 45.62; H, 7.36; N, 13.30. Found C, 45.74; H, 7.22; N, 13.37;
- [Mn^II^oxo_2_[14]aneN_4_)Cl_2_]·5H_2_O; Yield: 52%. Anal. Calcd. for C_10_H_22_O_2_N_4_Cl_2_Mn·5H_2_O: C, 26.92; H, 7.17; N, 12.56. Found C, 26.52; H, 7.02; N, 12.60.

#### 3.2.2. Dinuclear Complexes of Mn(III) and Mn(IV)

Dinuclear Mn(III) and Mn(IV) ions complexes were synthesized according to the previously described procedures [37,40].

The solution of Mn(ClO_4_)_2_·6H_2_O (10^−3^ mol) in ethanol (10 cm^3^) was added dropwise to the solution of ligands ([14]aneN_4_, Me_6_[14]aneN_4_, [12]aneN_4_ and [15]aneN_4_) (10^−3^ mol) in ethanol (10 cm^3^). The reaction mixture was then stirred for 1.5 h at room temperature. A small quantity of green-brown solid was formed. The reaction mixture was left in contact with the atmosphere for several days. A dark green crystalline product formed, which was collected by vacuum filtration, washed with ethanol and dried under vacuum at room temperature. As a result of this procedure, the following complexes were obtained:

- [Mn^III^Mn^IV^(*μ*-O)_2_([14]aneN_4_)_2_](ClO_4_)_3_·2H_2_O; Yield: 54%. Anal. Calcd. for C_20_H_48_O_14_N_8_Cl_3_Mn_2_·2H_2_O: C, 27.39; H, 5.93; N, 12.78. Found C, 27.33; H, 5.82; N, 12.71;
- [Mn^III^Mn^IV^(*μ*-O)_2_Me_6_[14]aneN_4_)_2_](ClO_4_)_3_·H_2_O; Yield: 45%. Anal. Calcd. for C_32_H_72_O_14_N_8_Cl_3_Mn_2_·2H_2_O: C, 37.42; H, 7.21; N, 10.91. Found C, 37.86; H, 7.05; N, 10.80;
- [Mn^III^Mn^IV^(*μ*-O)_2_([12]aneN_4_)_2_](ClO_4_)_3_·4H_2_O; Yield: 81%. Anal. Calcd. for C_16_H_40_O_14_N_8_Cl_3_Mn_2_·4H_2_O: C, 22.69; H, 5.67; N, 13.24. Found C, 23.91; H, 5.58; N, 13.20;
- [Mn^III^Mn^IV^(*μ*-O)_2_([15]aneN_4_)_2_](ClO_4_)_3_·2H_2_O; Yield: 51%. Anal. Calcd. for C_22_H_52_O_14_N_8_Cl_3_Mn_2_·2H_2_O: C, 29.20; H, 6.19; N, 12.39. Found C, 28.88; H, 6.02; N, 12.36.

Ligands: *N*-Me)_2_[14]aneN_4_, (*N*-Me)_4_[14]aneN_4,_ (*N*-Me)Me_2_py [14]aneN_4_ and oxo_2_[14]aneN_4_ do not form dinuclear complexes.

The chemical composition of the complexes was confirmed by colorimetric analysis, conductivity studies and complexometric titration with EDTA solutions of ascorbic acid and Eriochrome Black T indicator. Colorimetric analysis was based on the measurement of the absorbance of MnO_4_^−^ ions at 526 nm, obtained from the oxidation reaction of manganese ion complexes at lower oxidation states by IO_4_^−^ ions.

Ligands and complexes solutions were prepared immediately before measurements.

### 3.3. Other Reagents

MnClO_4_·6H_2_O and EDTA solution were from Fluka, MnCl_2_·4H_2_O was from Sigma-Aldrich. KIO_4_, Eriochrome Black T, ethanol, acetonitrile, KCl, KMnO_4_ and NaOH were purchased from POCH Gliwice, Poland. All of them were used without further purification.

### 3.4. Instruments and Procedures

UV-VIS spectra were obtained on a PU 8630 Philips spectrophotometer (Philips, Eindhoven, The Netherlands).

Infrared spectra were performed on a Specord M80 (Carl Zeiss, Jena, Germany) spectrometer using KBr pellets.

Conductance measurements were recorded on N-5772 TELECO conductancemeter (TELECO, Wrocław, Poland) at 298 K in ethanolic, acetonitrile and aqueous solutions of the complexes.

Spectroelectrochemistry measurements were performer using spectroelectrochemical instrument SPELEC DROPSENS (Metrohm DropSens, Oviedo, Spain) with reflection probe UV-VIS.

Cyclic voltammetry ware carried using AUTOLAB PGSTAT 10 (Eco Chemie BV, Utrecht, The Netherlands) in a three-electrode system. A glassy carbon disc electrode (GCE) was used as a working electrode. The SCE (MINERAL, Łomianki-Sadowa, Poland) connected to the bulk of the solution by a Luggin capillary was used as a reference electrode. A platinum plate was used as a counter electrode. Measurements were recorded in the presence of an aqueous solution of KCl (0.1 mol·dm^−3^) or HCl (0.1 mol·dm^−3^), as the supporting electrolyte. Solutions were deoxidised with argon before measurements.

Electrolyses with controlled potential were carried out at values corresponding to the respective anodic and cathodic peaks potential, using a spectroelectrochemical instrument, which allowed for recording spectra at any time during electrolysis.

Potentiometric measurements were carried out using microburette controlled by two-channel computer system (Cerko, Gdynia, Poland). For pH measurements (to determine *K* and *β*^II^) ligand (10^−3^ mol·dm^−3^), HCl (4 × 10^−3^ mol·dm^−3^, and/or MnCl_2_ (10^−3^ mol·dm^−3^) and a standard NaOH solution (4.5 × 10^−2^ mol·dm^−3^) were used. The exact concentration of the NaOH titrant and the CO_3_^2−^ content were determined using the Gran method. The titrated NaOH solutions contained ~1.4% CO_3_^2−^. A series of three titrations were performed for each ligand. The duration of one titration step was 40 s, with a permissible pH change of 0.018 pH units. In contrast, a series of five titrations was performed for each ligand in the presence of Mn^2+^, due to the longer equilibrium setting time. The duration of one titration step was 100 s, with a permissible pH change of 0.06 pH units. In both cases, 100 doses of titrant were added to each sample, to a titration fraction of ~4.5. Measurements covered a pH range of ~3 to ~11. pH measurements were carried out using a combined glass electrode with built-in Ag/AgCl as reference electrode (MINERAL, Łomianki-Sadowa, Poland), calibrated each day on three buffers, pH 4.01; 7.00 and 10.00. The slope of the calibration curve was usually within 57.6–57.9 range. For EMF measurements (to determine *β*^III^) ligand (10^−3^ mol·dm^−3^), HCl (10^−2^ mol·dm^−3^, MnCl_2_ (10^−2^ mol·dm^−3^) and a standard KMnO_4_ solution (c_1/5_ = 10^−1^ mol·dm^−3^) as oxidising agent, were used. Mn^3+^ ions in the tested cell containing KCl, MnCl_2_, ligand and HCl were produced following the addition of the oxidising agent, MnO_4_^−^. After each added portion of oxidant, once equilibrium was set, the potential of the tested redox system was measured using a combined electrode (Au, Ag/AgCl, MINERAL, Łomianki-Sadowa, Poland). The permissible EMF variation was set at 0.5 mV (20 readings per packet, packet interval 20 s). A series of five titrations were performed for each ligand in the presence of Mn^2+^. Each included _~_40 points. The low number of measurement points is due to difficulties in designing the Mn(II) oxidation experiment resulting from the tendency of Mn(III) to disproportionate. With more concentrated solutions of Mn(II) and ligand, this reaction could not be completely prevented, while with less concentrated oxidant solutions, the oxidation of Mn(II) to Mn(III) did not occur. The Au, Ag/AgCl electrode was tested prior to measurements against a solution containing equimolar amounts of K_4_[Fe(CN)_6_] (10^−3^ mol·dm^−3^) and K_3_[Fe(CN)_6_] (10^−3^ mol·dm^−3^). The measure of the suitability of the electrode was the potential corresponding to that of the reference electrode. Triple-distilled water was used to prepare the solutions. All solutions were deoxygenated with argon during the experiments to prevent possible oxidation (by O_2_) of Mn^2+^ ions in the presence of ligands and precipitation of MnO_2_. The low pH of the medium prevented the disproportionation reaction of the complexed Mn^3+^ ions. The formation constants of mononuclear complexes of Mn^3+^ ions with unsubstituted and *C*-substituted ligands were not determined, due to the high tendency to form dimers of Mn^3+^ and Mn^4+^ ions.

## 4. Conclusions

In the presence of unsubstituted and *C*-substituted tetraazamacrocycles, in neutral mediaMn^2+^ form dinuclear Mn^3+^ and Mn^4+^ complexes; in acidic media mononuclear Mn^3+^ complexes. The electron spectra of the mononuclear *cis*- and *trans* structures are characterised by the same type of bands shifted with respect to each other. IR spectra differ in the number of bands within the ranges characteristic for deformation vibrations of the amine and methylene groups. The electron and IR spectra of the dinuclear complexes show the same features, independent of the ligand type. Mononuclear Mn^3+^ complexes oxidise very readily to dimers, a process preceded by *trans–cis* isomerisation at the level of the Mn^2+^ complexes. These ligands significantly stabilise the high oxidation state of the manganese ion by the fact that Mn^3+^ ions in complexes with these ligands do not disproportionate and the very low formal potentials (*E_f_^0^*) of the *trans*-[Mn^III^LCl_2_]^+^/*trans*-[Mn^II^LCl_2_] and [Mn^III^Mn^IV^(*μ*-O)_2_L_2_]^3+^/[Mn^III^Mn^III^(*μ*-O)_2_L_2_]^2+^ redox systems.

Among the *N*-substituted tetraazamacrocycles and oxo_2_[14]aneN_4_ ligands. Under current conditions, these complexes oxidise to mononuclear Mn^3+^ ion complexes according to the EC mechanism and undergo disproportionation. These ligands stabilise the high oxidation state of the manganese ion to a much lesser extent, as indicated by the much higher *E_f_*^0^ value and the fact of disproportionation. Among these complexes, Mn^3+^ ions are best stabilised by (*N*-Me)Me_2_py [14]aneN_4_, most likely as a result of the predominantly inductive over mesomeric effect and the weaker solvation of the ligand with lower hydrophilicity, as indicated by the lowest values of the disproportionation constant, *k*_1_ and *E_f_*^0^, and the highest value of the stability constant (*β*^III^). In contrast, Mn^3+^ ions are least stabilised by oxo_2_[14]aneN_4_, due to the reduced electron density of the two amide N atoms. The relation of *k*_1_ and *E_f_*^0^ as well as (*β*^III^) is reversed.

## Figures and Tables

**Table 1:** Values of disproportionation constants (*k*_1_) and stability constants of Mn^2+^ and Mn^3+^ complexes.

Ligand	*k*_1_	log*β^(^*^II)^	log*β*^(III)^
[14]anN_4_	-	11.06 ± 0.06	-
Me_6_[14]anN_4_	-	11.12 ± 0.08	-
[12]anN_4_	-	11.74 ± 0.06	-
[15]anN_4_	-	10.81 ± 0.07	-
(*N*-Me)_2_[14]anN_4_	3.19 ± 0.14	11.41 ± 0.06	22.68 ± 0.12
(*N*-Me)_4_[14]anN_4_	2.87 ± 0.16	11.35 ± 0.06	22.14 ± 0.10
(oxo)_2_[14]anN_4_	4.52 ± 0.12	10.26 ± 0.07	18.27 ± 0.12
(*N*-Me)Me_2_py [14]anN_4_	1.01 ± 0.14	11.98 ± 0.08	24.06 ± 0.11

## Data Availability

Data are contained within the article and Supplementary Material.

## Supplementary Materials

The following supporting information can be downloaded at: https://www.mdpi.com/article/10.3390/molecules30081860/s1, Figure S1: UV VIS NIR spectra of 10^−3^ mol·dm^−3^ ligands and complexes in 0.1 mol·dm^−3^ KCl, 0.093 cm cell: yellow line—(*N*-Me)_2_[14]aneN_4_; blue line—[Mn^II^((*N*-Me)_2_[14]aneN_4_)Cl]Cl; red line—(*N*-Me)Me_2_py [14]aneN_4_; black line—[Mn^II^((*N*-Me)Me_2_py [14]aneN_4_)Cl]Cl; Figure S2: Cyclic voltammogram of 10^−3^ mol·dm^−3^ [Mn^III^Mn^IV^(*μ*-O)_2_Me_6_[14]aneN_4_)_2_](ClO_4_)_3_ in 0.1 mol·dm^−3^ KCl, 50 mV·s^−1^, GCE vs. SCE; Figure S3: UV VIS spectra of 10^−3^ mol·dm^−3^ [Mn^III^Mn^IV^(*μ*-O)_2_Me_6_[14]aneN_4_)_2_](ClO_4_)_3_ in 0.1 mol·dm^−3^ KCl; before electrolysis with controlled working electrode potential—black line; after electrolysis at 0 V—blue line; after electrolysis at 1 V—red line; 0.995 cm cel; Figure S4: Cyclic voltammogram of 10^−3^ mol·dm^−3^ *cis*-[Mn^III^([12]aneN_4_)Cl_2_]Cl in 0.1 mol·dm^−3^ HCl, 50 mV·s^−1^, GCE vs. SCE; Figure S5: Cyclic voltammogram of 10^−3^ mol·dm^−3^ *trans*-[Mn^III^Me_6_[14]aneN_4_)Cl_2_]Cl in 0.1 mol·dm^−3^ HCl, 50 mV·s^−1^, GCE vs. SCE; Figure S6: Cyclic voltammograms of 10^−3^ mol·dm^−3^ *trans*-[Mn^III^Me_6_[14]aneN_4_)Cl_2_]Cl in 0.1 mol·dm^−3^ KCl; red line—1st cycle; blue line—3rd cycle; black line—10th cycle; 50 mV·s^−1^, GCE vs. SCE; Figure S7. Partition curve of free Mn^2+^ ions and MnL^2+^ complex against pH, L—(*N*-Me)Me_2_py [14]aneN_4_; Table S1: Resalt of UV VIS NIR spectra mononuclear Mn^3+^ complexes in water solution; Table S2: Resalt of UV VIS NIR spectra dinuclear Mn^3+^ and Mn^4+^ complexes in water solution; Table S3: Resalt of IR spectra mononuclear manganese complexes in KBr pellet; Table S4: Resalt of IR spectra dinuclear manganese complexes in the 500–950 cm^−1^ range, in KBr pellet; Table S5: Formal potentials of redox systems [Mn^III^LCl_2_]^+^/[Mn^II^LCl_2_] and [Mn^III^Mn^IV^(*μ*-O)_2_L_2_]^3+^/[Mn^III^Mn^III^(*μ*-O)_2_L_2_]^2+^ (L—ligand) in water solution, GCE vs. SCE; Table S6. Protonation constants of the investigated tetraazamacrocycles, T = 293 K, *μ* = 0.1(10^−1^ mol·dm^−3^ KCl).
