# Supplementary material for: Influence of the Type of Macrocycle on the Stabilisation of the High Oxidation State of the Manganese Ion and Electrode Processes
Source: Molecules. 2025 Apr 21;30(8):1860. doi: 10.3390/molecules30081860 (PMC12029323; doi:10.3390/molecules30081860)
Supplement: Supplementary file 1 [file molecules-30-01860-s001.zip › molecules-3564406-supplementary.pdf]

Supplementary materials

Influence of the type of macrocycle on the stabilisation of the high oxidation state of the manganese ion

D. Tomczyk, S. Skrzypek, P. Seliger

Figures

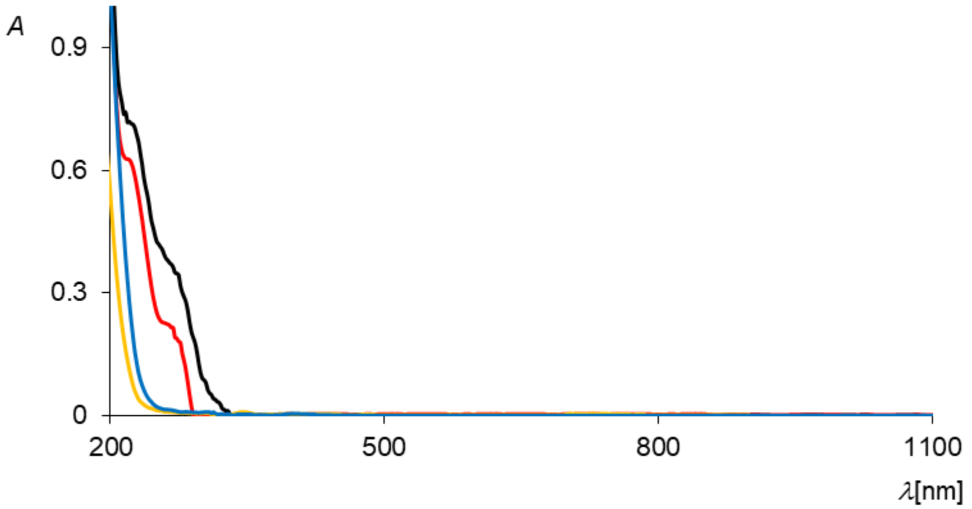

Figure S1. UV VIS NIR spectra of 10^{-3} mol·dm^{-3} ligands and complexes in 0.1 mol·dm^{-3} KCl, 0.093 cm cell: yellow line - (*N*-Me)$_2$[14]aneN$_4$; blue line - [Mn^{II}((*N*-Me)$_2$[14]aneN$_4$)Cl]Cl; red line - (*N*-Me)Me$_2$py[14]aneN$_4$; black line - [Mn^{II}((*N*-Me)Me$_2$py[14]aneN$_4$)Cl]Cl.

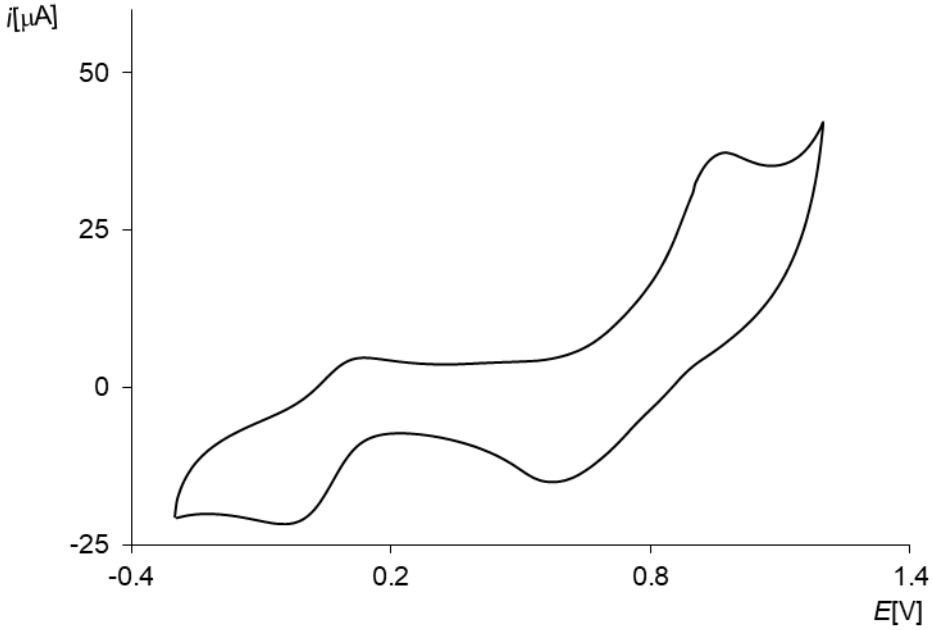

Figure S2 Cyclic woltammogram of 10^{-3} mol·dm^{-3} [Mn^{III}Mn^{IV}(μ-O)$_2$[15]aneN$_4$)$_2$](ClO$_4$)$_3$ in 0.1 mol·dm^{-3} KCl, 50 mV·s^{-1}, GCE vs SCE.

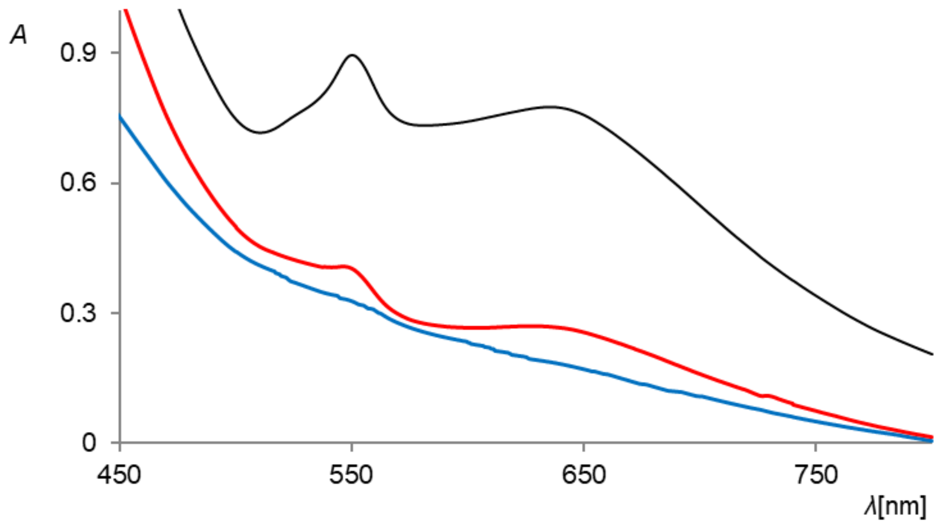

Figure S3 UV VIS spectra of 10^{-3} mol·dm^{-3} $[Mn^{III}Mn^{IV}(\mu\text{-}O)_2Me_6[14]aneN_4)_2](ClO_4)_3$ in 0.1 mol·dm^{-3} KCl; before electrolysis with controlled working electrode potential - black line; after electrolysis at 0 V - blue line; after electrolysis at 1 V – red line; 0.995 cm cell.

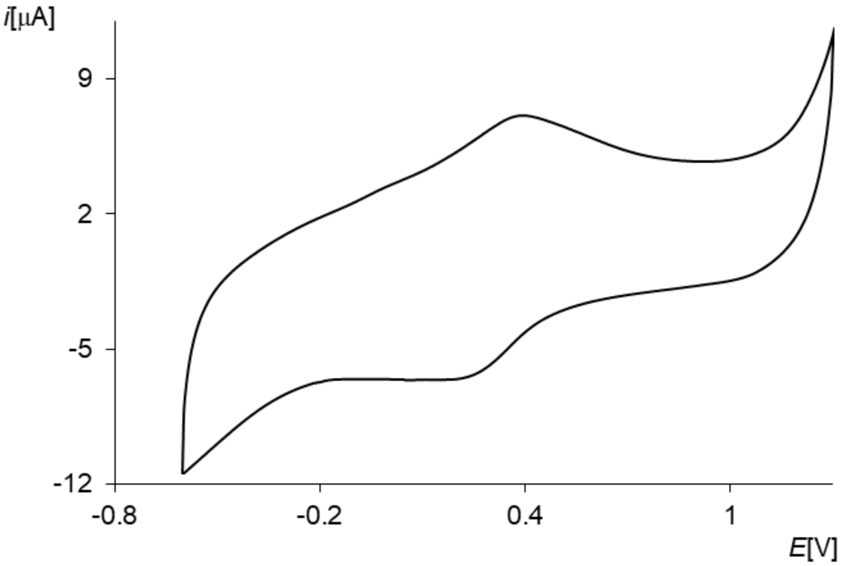

Figure S4. Cyclic woltammogram of 10^{-3} mol·dm^{-3} *cis*-$[Mn^{III}([12]aneN_4)Cl_2]Cl$ in 0.1 mol·dm^{-3} HCl, 50 mV·s^{-1}, GCE vs SCE.

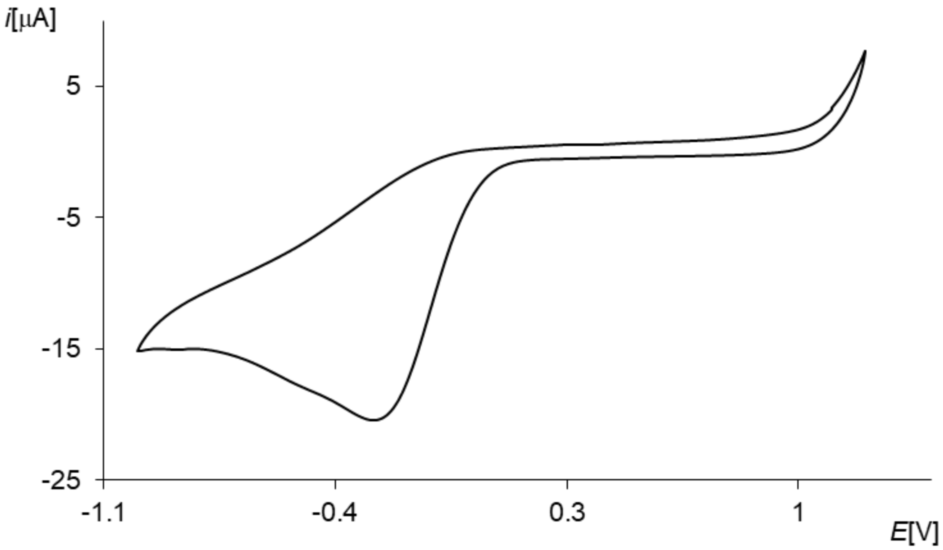

Figure S5. Cyclic woltammogram of 10^{-3} mol·dm^{-3} *trans*-[$Mn^{III}Me_6$[14]aneN_4)Cl_2]Cl in 0.1 mol·dm^{-3} HCl, 50 mV·s^{-1}, GCE vs SCE.

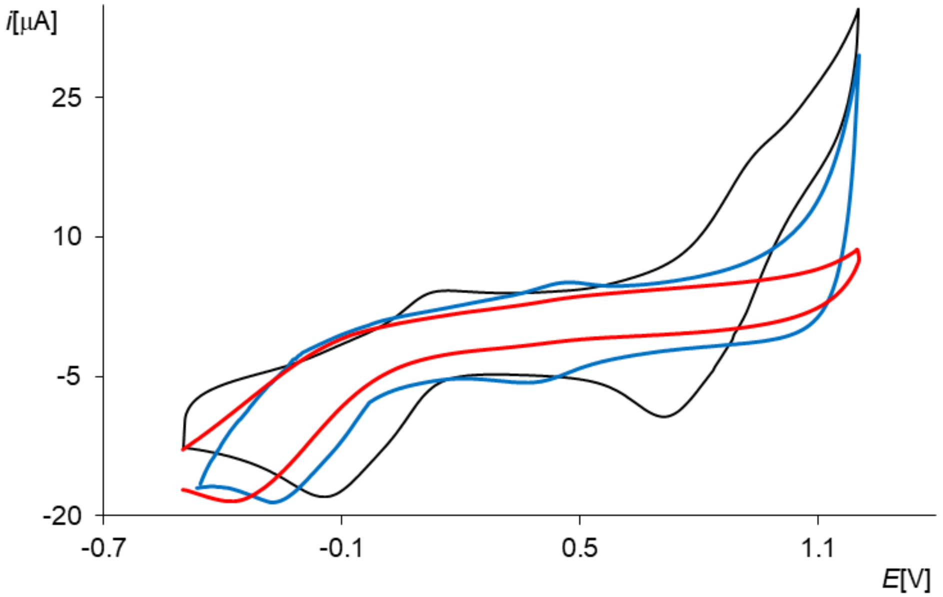

Figure S6. Cyclic woltammograms of 10^{-3} mol·dm^{-3} *trans*-[$Mn^{III}Me_6$[14]aneN_4)Cl_2]Cl in 0.1 mol·dm^{-3} KCl; red line – 1st cycle; blue line – 3rd cycle; black line – 10th cycle; 50 mV·s^{-1}, GCE vs SCE.

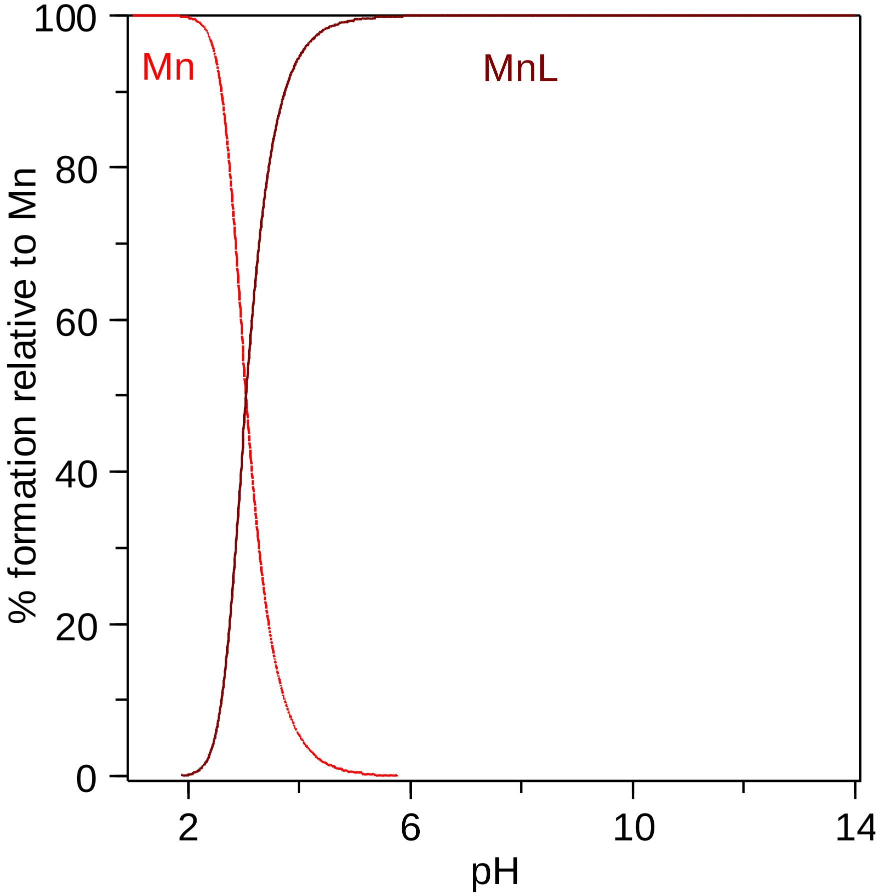

Figure S7. Partition curve of free Mn^{2+} ions and MnL^{2+} complex against pH, L – (*N*-Me)Me_2py[14]aneN_4.

Tables

Table S1. Resalt of UV VIS NIR spectra mononuclear Mn^{3+} complexes in wather solution..

Complex•	$\lambda_1(\varepsilon_1)$	$\lambda_2(\varepsilon_2)$	λ_3
trans-[Mn^{III}([14]anN_4)Cl_2]Cl	271 (10160)	342 (1980)	710
trans-[Mn^{III}(Me_6[14]anN_4)Cl_2]Cl	298 (2020)	350 (760)	700
cis-[$Mn^{III(}$[12]anN_4)Cl_2]Cl	228 (14580)	304 (4300)	698
trans-[Mn^{III}([15]anN_4)Cl_2]Cl	312 (7630)	398 (2820)	712

Table S2. Resalt of UV VIS NIR spectra dinuclear Mn^{3+} and Mn^{4+} complexes in wather solution.

Complex	λ_1	$\lambda_2(\varepsilon_2)$	$\lambda_3(\varepsilon_3)$
[$Mn^{III}Mn^{IV}$(μ-O)$_2$([14]anN_4)$_2$](ClO_4)$_3$	540	552 (760)	642 (770)
[$Mn^{III}Mn^{IV}$(μ-O)$_2$(Me_6[14]anN_4)$_2$](ClO_4)$_3$	544	556 (730)	650(710)
[$Mn^{III}Mn^{IV}$(μ-O)$_2$([12]anN_4)$_2$](ClO_4)$_3$	542	554 (710)	658 (900)
[$Mn^{III}Mn^{IV}$(μ-O)$_2$([15]anN_4)$_2$](ClO_4)$_3$	548	560 (740)	654 (730)

Table S3. Resalt of IR spectra mononuclear manganese complexes in KBr pellet.

complex*	cm^{-1}					
	valence vibrations			deformation vibrations		
	O-H, N-H	CH_2, CH_3,	C-N	CH_2, CH_3	CH_2	N-H
[14]anN_4<br>*trans*-[Mn^{III}([14]anN_4)Cl_2]Cl	3439m;<br>3428s;	2869s;<br>2855s;	1129s<br>1125m	1332m;<br>1316m;	793m<br>807w	832s<br>880
Me_6[14]anN_4<br>*trans*-[Mn^{III}(Me_6[14]anN_4)Cl_2]Cl	3436m;<br>3424s;	2891s, 2710w;<br>2874m, 2702w;	1121m<br>1114m	1380m, 1348m;<br>1369w, 1332w;	798m<br>807	831w<br>881
[12]anN_4<br>*cis*-[Mn^{III}([12]anN_4)Cl_2]Cl	3447m;<br>3420s;	2891s;<br>2870m;	1129s<br>1123m	1350m;<br>1343m;	814m<br>823m, 791m	856m<br>912m, 877w, 859m
[15]anN_4<br>*trans*-[Mn^{III}([15]anN_4)Cl_2]Cl	3439m;<br>3420s;	2869s;<br>2858m;	1129s<br>1127w	1339m;<br>1331w;	800m<br>811w	841w<br>889w, 879m

	valence vibrations						deformation vibrations
	O-H, N-H;	CH_3, CH_2, =CH;	C=O;	C=N;	C=C;	C-N	CH_3, CH_2; =CH N-H
(*N*-Me)$_2$[14]anN_4<br>[Mn^{II}((*N*-Me)$_2$[14]anN_4)Cl]Cl	3411m;<br>3402s;	2964m, 2868m;<br>2961m, 2864m;				1128w<br>1121w	1383w, 1349w, 856w<br>810w;<br>1383w, 1346w, 848w<br>804w;
(*N*-Me)$_4$[14]anN_4<br>[Mn^{II}((*N*-Me)$_4$[14]anN_4)Cl]Cl	3385w;<br>3385s;	2964m,2855s;<br>2959m, 2855m;				1132m<br>1124w	1390w, 1336w,<br>784w<br>1389w, 1336w,<br>779w
okso$_2$[14]anN_4<br>[Mn^{II}(okso$_2$[14]anN_4)Cl_2]	3335m, 3280w;<br>3331s, 3277s;	2874m;<br>2871m;	1679s;<br>1652s;			1560s, 1172m<br>1545s, 1155m	1360m, 833w; 889w<br>1362m, 828w; 897w
(*N*-Me)Me_2py[14]anN_4<br>[Mn^{II}((*N*-Me)Me_2py[14]anN_4)Cl]Cl	3420m;<br>3416s;	2960m;<br>2957m;	1674s;<br>1652s;		1450m,<br>1540s;<br>1445m<br>1537s;	1192w<br>1179w	1315w; 750w, 859w<br>684m;<br>1312w; 746w, 848w<br>681m

Table S4. Resalt of IR spectra dinuclear manganese complexes in the 500 – 950 cm^{-1} range, in KBr pellet.

Kompleks•	cm^{-1}		
	δ_{-CH_2-} (790-830)	δ_{-NH-}	$[Mn^{III}(\mu\text{-}O)_2Mn^{IV}]^{3+}$
$([Mn^{III}Mn^{IV}(\mu\text{-}O)_2([14]anN_4)_2](ClO_4)_3$	806(w), 794(w)	924(w), 860(w), 851(w)	680(s)
$[Mn^{III}Mn^{IV}(\mu\text{-}O)_2(Me_6[14]anN_4)_2](ClO_4)_3$	803(w), 790(w)	930(w), 861(w), 855(w)	684(s)
$[Mn^{III}Mn^{IV}(\mu\text{-}O)_2([12]anN_4)_{22}](ClO_4)_3)$.	824(w), 805(m)	918(w), 862(w), 859(w)	688(s)
$[Mn^{III}Mn^{IV}(\mu\text{-}O)_2([15]anN_4)_2](ClO_4)_3$	804(w), 792(w)	911(w), 871(w), 852(w)	685(s)

Table S5. Formal potentials of redox systems $[Mn^{III}LCl_2]^+/[Mn^{II}LCl_2]$ and $[Mn^{III}Mn^{IV}(\mu\text{-}O)_2L_2]^{3+}/[Mn^{III}Mn^{III}(\mu\text{-}O)_2L_2]^{2+}$ (L - ligand) in wather solution, GCE vs SCE.

Complexes		E^0_f [V] vs SCE
trans-$[Mn^{III(}[14]anN_4)Cl_2]Cl$		-0.16±0.03
trans-$[Mn^{III}(Me_6[14]anN_4)Cl_2]Cl$		-0.10±0.02
cis-$[Mn^{III(}[12]anN_4)Cl_2]Cl$		0.34±0.03
trans-$[Mn^{III}([15]anN_4)Cl_2]Cl$		-0.04±0.02
$[Mn^{III}Mn^{IV}(\mu\text{-}O)_{2(}[14]anN_4)_2](ClO_4)_3$		0.05±0.03
$[Mn^{III}Mn^{IV}(\mu\text{-}O)_2(Me_6[14]anN_4)_2](ClO_4)_3$		0.02±0.03
$[Mn^{III}Mn^{IV}(\mu\text{-}O)_2[12]anN_{42}](ClO_4)_3$		0.01±0.03
$[Mn^{III}Mn^{IV}(\mu\text{-}O)_2([15]anN_4)_2](ClO_4)_3$		0.07±0.03
$[Mn^{II}((N\text{-}Me)_2[14]anN_4)Cl]Cl$		0.84±0.02
$[Mn^{II}((N\text{-}Me)_4[14]anN_4)Cl]Cl$		0.87±0.02
$[Mn^{II}(okso_2[14]anN_4)Cl_2]$		0.89±0.03
$[Mn^{II}((N\text{-}Me)Me_2py[14]anN_4)Cl]Cl$		0.80±0.02

Table S6. Protonation constants of the investigated tetraazamacrocycles, T= 293 K, μ = 0.1(10^{-1} mol·dm^{-3} KCl).

Ligand	logK_1	logK_2	logK_3	logK_4
[14]anN_4	10.95±0.12	10.08±0.06	2.61±0.12	1.60
Me_6[14]anN_4	9.88±0.10	6.42±0.05	2.54±0.14	2.09±0.18
[12]anN_4	10.34±0.10	9.73±0.06	2.09±014.	<1
[15]anN_4	10.82±0.12	9.91±0.08	5.06±0.15	3.02±0.20
(*N*-Me)$_2$[14]anN_4	10.85±0.10	9.64±0.05	2.17±0.12	<1
(*N*-Me)$_4$[14]anN_4	9.74±0.11	9.17±0.05	2.66±0.12	2.27±0.18
(okso)$_2$[14]anN_4	9.26±0.10	5.40±0.06	-	-
(*N*-Me)Me_2py[14]aneN_4	9.05±0.12	6.30±0.06	3.21±0.14	1.81±0.20
